# Prospective Comparative Quality Control Study of a Novel Gravity-Driven Hollow-Fiber Whole Blood Separation System for the Production of Canine Blood Products

**DOI:** 10.3389/fvets.2019.00149

**Published:** 2019-05-17

**Authors:** Hendrik Lehmann, Esther Hindricks, Esther Maria Hassdenteufel, Andreas Moritz, Natali Bauer

**Affiliations:** ^1^Department of Veterinary Clinical Sciences, Small Animal Clinic, Justus-Liebig-University Giessen, Giessen, Germany; ^2^Department of Veterinary Clinical Sciences, Clinical Pathology and Clinical Pathophysiology, Justus-Liebig-University Giessen, Giessen, Germany

**Keywords:** polyethersulfone, gravity-driven hollow-fiber, leukodepletion, blood product quality, coagulation

## Abstract

The aim of this prospective study was to compare quality of blood products produced either by a novel gravity-driven hollow-fiber separation system (HF) or by centrifugation (C). Whole blood was obtained from 31 healthy non-greyhound canine blood donors and separated into fresh frozen plasma and packed red blood cells using either HF or C in a university teaching hospital. Red blood cell (RBC) count, albumin and fibrinogen concentration, prothrombin time (PT), activated partial thromboplastin time (aPTT) and coagulation factor activity (FV, FVIII), von Willebrand Factor (vWF), and antithrombin activity were assessed. Plasma obtained with the HF showed a significantly higher median PT (9.4 vs. 7.9 s, *P* = 0.0006) and aPTT (14.9 vs. 13.1 s, *P* = 0.0128) than plasma prepared with C. Lower albumin (21.7 vs. 23.5 g/l, *P* = 0.0162) and fibrinogen (1.0 vs. 1.5 g/l, *P* = 0.0005) concentrations and activities of FV (105 vs. 114%, *P* = 0.0021) and antithrombin (104 vs. 117%, *P* = 0.0024) were seen in blood products obtained with the HF. In contrast, vWF was not affected by the method of plasma separation. Compared to HF, RBC count as well as hematocrit were not significantly higher (8.0 vs. 8.9 10^12^/l, *P* = 0.1308; 0.57 vs. 0.62 l/l, *P* = 0.0736) when blood products were prepared with C. In conclusion, higher quality of blood products especially regarding coagulation parameters and RBCs was achieved by using C compared to HF. Despite the statistical significances, however, the clinical relevance has to be further elucidated. Nevertheless, HF provides an alternative to produce blood products if a centrifuge is not available.

## Introduction

Transfusion of blood components gained increasing importance in veterinary medicine (1, 2). Preparation of blood product components is expensive and time consuming as specific devices for separation such as a centrifuge are needed (3). The presence of commercial veterinary blood banks in the USA improved the availability of blood components (4, 5). However, in other countries, commercial blood banks are scarce. Therefore, blood banks owned by veterinary practitioners became essential in the veterinary field. However, if small quantities of blood products are required in smaller veterinary clinics or practices, the initial costs for separation using a centrifugation protocol are high.

**Figure 1:**
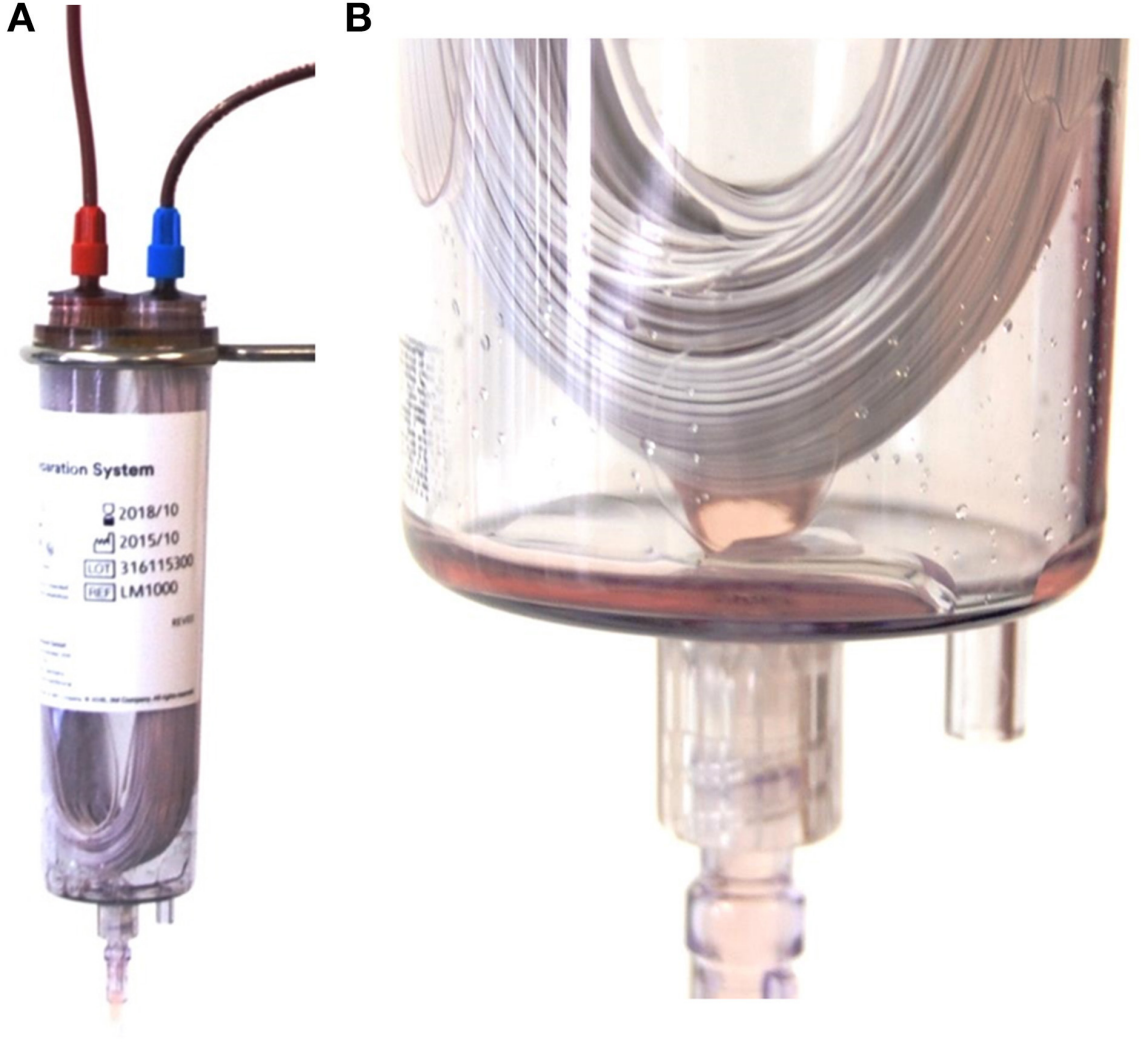
**(A)** Composition of the hollow-fiber system (HF) showing the U shaped polyethersulfone fibers in a plastic chamber and connected with the whole blood bag (not shown) with the red Luer lock connector (left on the top of the chamber) and the packed red blood cell bag (not shown) with the blue luer lock connector (right on top of the plastic chamber). **(B)** Detailed image of the U shaped fibers at the bottom of the plastic chamber. Picture taken during the process of plasma separation. Small droplets of plasma can be seen leaving the fibers and entering the plasma bag (not shown) via a luer lock connection (bottom of the picture).

For people, alternative systems have been developed using gravity without the need for heavy centrifuges requiring electric power supply or for larger centrifuges high voltage power (three-phase electric power). They allow the preparation of life-saving blood products in the military field or zones with small blood donation facilities (6–10). These systems consist of several hundreds to thousands of hollow-fibers of various types of materials such as polyethylene (11), polyethersulfone (12–14), or polypropylene (15), through which the blood flow is achieved by gravitation. The hollow-fibers are characterized by different wall thicknesses, inner diameters, and pore sizes, respectively. They are built into a plastic chamber forming a U shape exemplarily shown in Figure 1.

At the distal loop of the fibers, plasma is expressed through the pores and collected inside the chamber, which is further connected with the plasma bag. The remaining plasma poor blood which cannot pass the fiber pores is further driven to an erythrocyte storage bag connected to the system, the packed red blood cell bag (pRBC). In human medicine, the quality of the obtained blood products is regulated by national and international guidelines (16). In the veterinary field, scarce regulations are available. There are national guidelines for the collection, storage, transport and administration of blood and blood products in some countries e.g., in Germany [later referred to as German veterinary guideline for blood products (17)]. Overall, guidelines regulate the quality of the produced pRBC and fresh frozen plasma (FFP) (17).

Based on the German veterinary guideline for blood products (17), quality of the FFP is fulfilled if 50% of the initial coagulation Factor VIII (FVIII) activity is preserved and if it contains <1 × 10^9^/l white blood cells, <30 × 10^9^/l thrombocytes, and <3 × 10^9^/l erythrocytes.

In the pRBC, phosphate (Phos) concentration and lactate dehydrogenase (LDH) activity are of interest as their increase reflects potential hemolysis. Hemolysis should be <0.8% at the end of storage. Quality of the pRBC is further evaluated by measuring the hemoglobin concentration, hematocrit value, and red blood cell count. Potassium (K) concentration is a marker of ATP depletion during storage.

Despite the lack of international veterinary guidelines, some text books (18, 19) offered a non-binding guideline for sampling, preparation, storage, and administration of blood products based on the literature.

To the authors' knowledge, two commercial gravity-driven separation systems are currently available and have been recently evaluated in people (8, 13). It was shown that the use of hollow-fiber (HF) systems causes a significant reduction in Factor V (FV), Factor VIII (FVIII), Factor XI, and fibrinogen concentration. Due to the decrease in FVIII, these plasma products do not fulfill international criteria for plasma quality (16) making HF blood separation systems a less ideal alternative for blood product separation in human medicine (8, 13).

However, to the authors' knowledge there is currently no veterinary study evaluating quality of blood products prepared with gravity-driven separation systems. Thus, the aim of this study was to assess quality of blood products (plasma and pRBC) obtained with a novel HF system in comparison to the traditional method, the centrifuge.

Hypothesis of this study was that the quality of blood products produced by a novel gravity-driven HF separation system is lower than in products generated by centrifugation but still fulfills quality requirements recommended previously for veterinary blood products (17).

## Materials and Methods

### Study Design

The prospective study was performed between February and June 2017.

Dogs were recruited from the pre-established blood donation program of a veterinary teaching hospital including client owned dogs.

The blood donation program was ethically approved by the regional council (No V 54 - 19 c 20 15 h 02 GI 18/7 No. A 24/2017) and written owner's consent was given.

Criteria for inclusion to the donor program were based on the German veterinary guideline for blood products (17). Briefly, dogs with a body weight >20 kg were included if history and clinical examination were unremarkable and there was no traveling history in the Mediterranean area or other continents. If clinical examination was unremarkable, a 20 G catheter^1^ was aseptically placed in the cephalic or lateral saphenous vein by a licensed veterinary technician or veterinarian and blood was taken for laboratory examination routinely done prior to each blood donation. Overall, a total of four tubes coated with ethylenediamine tetraacetic acid (EDTA), two lithium heparin tubes, two plain tubes, and four tubes containing 3.2% sodium citrate were drawn, whereby each tube had a volume of 1.3 ml.

Routine laboratory examination included blood typing (if not performed before) using a commercially available quick test^2^, as well as hematological (CBC) and blood chemical examination. Biochemical evaluation included assessment of electrolytes, urea, creatinine, glucose, total protein, albumin, globulin, and C reactive protein. In case of no clinically significant abnormalities, blood donation was performed and the dog was enrolled in the study. Immediately after sampling, the citrated tubes were centrifuged^3^ at 3,000 G for 5 min and the supernatant was removed and centrifuged again for 5 min at 3,000 G. Citrated plasma was then stored at −80°C until coagulation analysis.

After blood donation, the dogs were assigned randomly to the groups for hollow-fiber (HF) separation and centrifugation (C) method, respectively such that an approximately equal number of blood donors was achieved in each group whereby a special technique of randomization was not used (20). In both groups, the same procedure of blood donation and the same blood bags^4^ for transfer and storage of blood products were used.

### Donation

A triple bag system^4^ consisting of a 17G needle for puncturing the jugular vein, a donation bag with 63 ml Citrate-Phosphate-Dextrose (CPD) anticoagulant, a red blood cell bag containing 100 ml Saline Adenine Glucose-Mannitol-Solution (SAG-M), an empty plasma bag as well as a leukocyte depletion filter^5^, and a Y-connector with screwable luer lock connectors were used for blood collection. The triple blood bag system was provided by the manufacturer and was used for both groups. Donation was performed using gravity until the blood collection mixer^6^ showed the targeted amount of 10–20 ml/kg of donated blood considered to be well tolerated by the donor (21) so that a final amount of 400–500 ml was approximated (**Figure 3**).

During donation, the dogs received a continuous infusion of 10 ml/kg/h buffered crystalloid solution^7^ administered with a volumetric infusion pump^8^ via the venous catheter to account for any possible hemodynamic effects of blood donation. Every dog received the same rate of crystalloids for the duration of donation process. Donated blood was further gently mixed for a few minutes and allowed to rest at room temperature for a maximum of 1 h. Afterwards, the exact weight of the whole blood bag (WB) was determined and converted into volume whereby the following correction factors were applied: Whole blood: 1,050 g/ml, plasma: 1,030 g/ml, and packed red blood cells: 1,070 g/ml (22, 23), respectively.

### Separation Process

#### Hollow-Fiber Group

**Figure 2:**
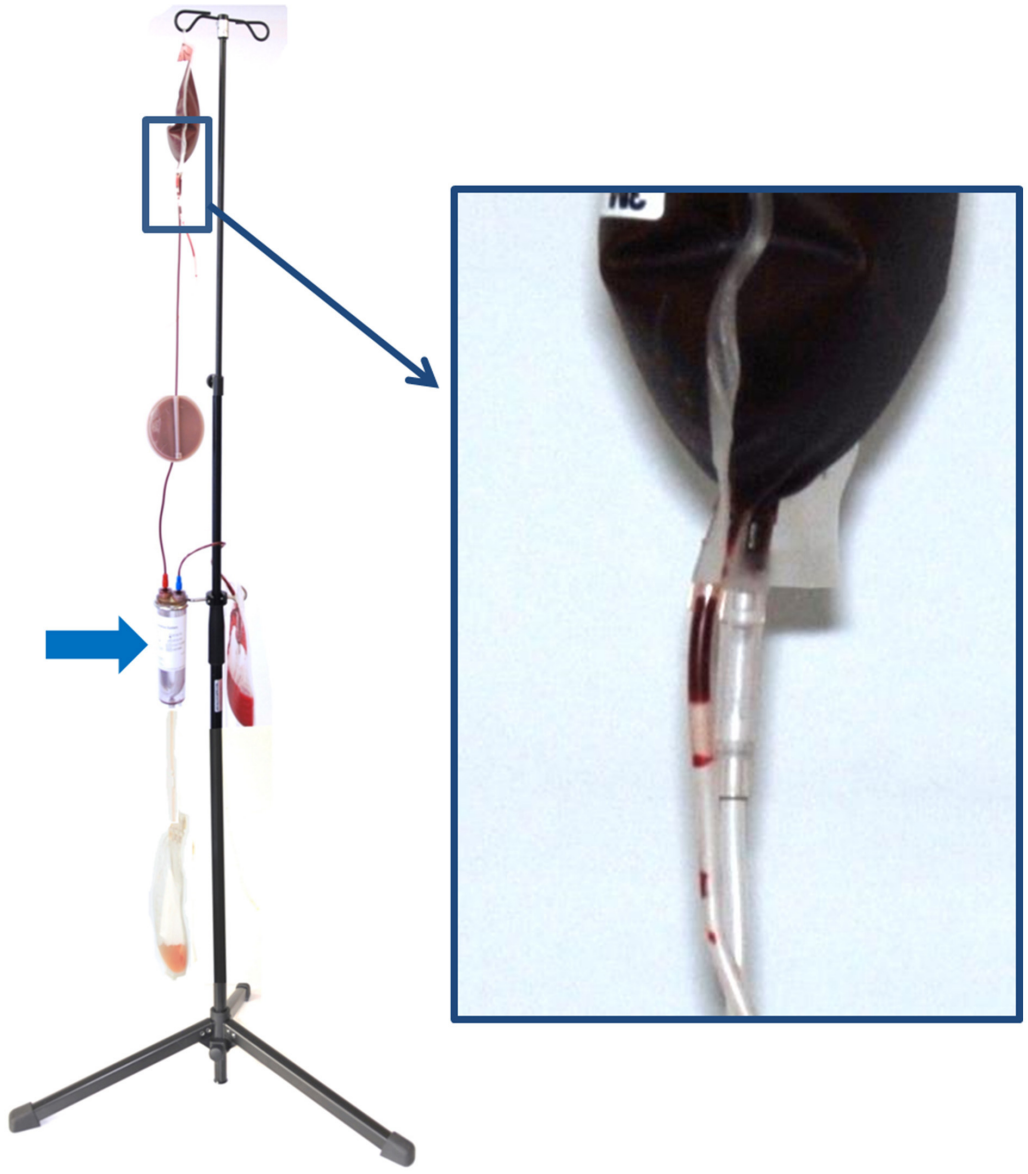
Configuration of the hollow-fiber system (HF) set up on a stand for gravity-driven blood separation. On top, there is the whole blood bag (WB), which is connected via the in-line leukocyte depletion filter (arrow) to the hollow-fiber system (HF). During blood separation, plasma leaving the U-shaped fibers of the HF is collected in the plasma bag (P) and the remainder erythrocytes are stored in the packed red blood cell bag (pRBC). The inset shows the breaking valve of the whole blood bag, which is broken to initiate the process of blood separation.

The WB was hung up at the stand provided by the manufacturer for separation process (Figure 2).

An in-line leukodepletion filter^5^ with a screwable luer lock connector was implemented (by the manufacturer)^4^ into the connection line of the WB. It was attached to the HF filtration polyethersulfone device (MicroPES® TF 10 capillary membrane)^9^.

The system was further connected to the bag containing SAG-M and to the empty bag for plasma collection after being processed through the filter (Figures 1, 2).

The HF filter consists of polyethersulfone with 100 μm ± 25 wall thickness, an inner diameter of 300 ± 40 μm and a pore size of 0.5 μm (compare Figure 2). The filter is delivered with a 25–35 ml saline priming volume as a dry filter would cause an extremely rapid absorption of plasma resulting in a hemolysis of RBCs (24).

At the distal loop of the fiber filter system, plasma was pressed from the system by gravitation through the membrane. By breaking the valve (Figure 2, inlet), the separation process was started and the time of beginning and finishing noted. After the separation process, the filter system was not rinsed with saline to yield the remaining RBCs as it was not recommended by the manufacturer.

Plasma (P) and erythrocyte bag (pRBC) were disconnected and gently mixed.

#### Centrifugation Group

Prior to separation via centrifugation, leukodepletion was performed to make the procedure comparable to the HF group. For leukodepletion, the WB with the in-line Leukodepletion filter^5^ was connected via the luer lock connector to an additional plain, empty plasma bag and the separation process was started by breaking the valve of the WB (as shown in Figure 2 for the HF) and duration of complete separation process was noted. When leukodepletion was finished, the WB was then prepared for centrifugation by placing it upwardly into the centrifugation cup and a further cup of exactly equal weight was prepared and balanced.

Centrifugation was performed for 20 min (plus 10 min deceleration time) at 1,976 G using a commercial centrifuge designed for blood separation^10^.

After centrifugation, the WB containing the now spun down erythrocytes and plasma was connected to the dedicated plasma bag. Plasma was separated from erythrocytes by applying pressure on the WB with a manual plasma press^11^. After plasma was harvested, the remainder red blood cells, i.e., the erythrocyte concentrate was transferred to the dedicated SAG-M bag (later referred to as pRBC) by gravitation. Both bags were gently mixed afterwards. The duration of the whole separation process using the centrifuge was recorded and included the time required for leukodepletion, preparing the centrifugation cups, the centrifugation time itself as well as manual expression of plasma into the plasma bag.

### Sampling and Sample Processing for Quality Control of Blood Products

**Figure 3:**
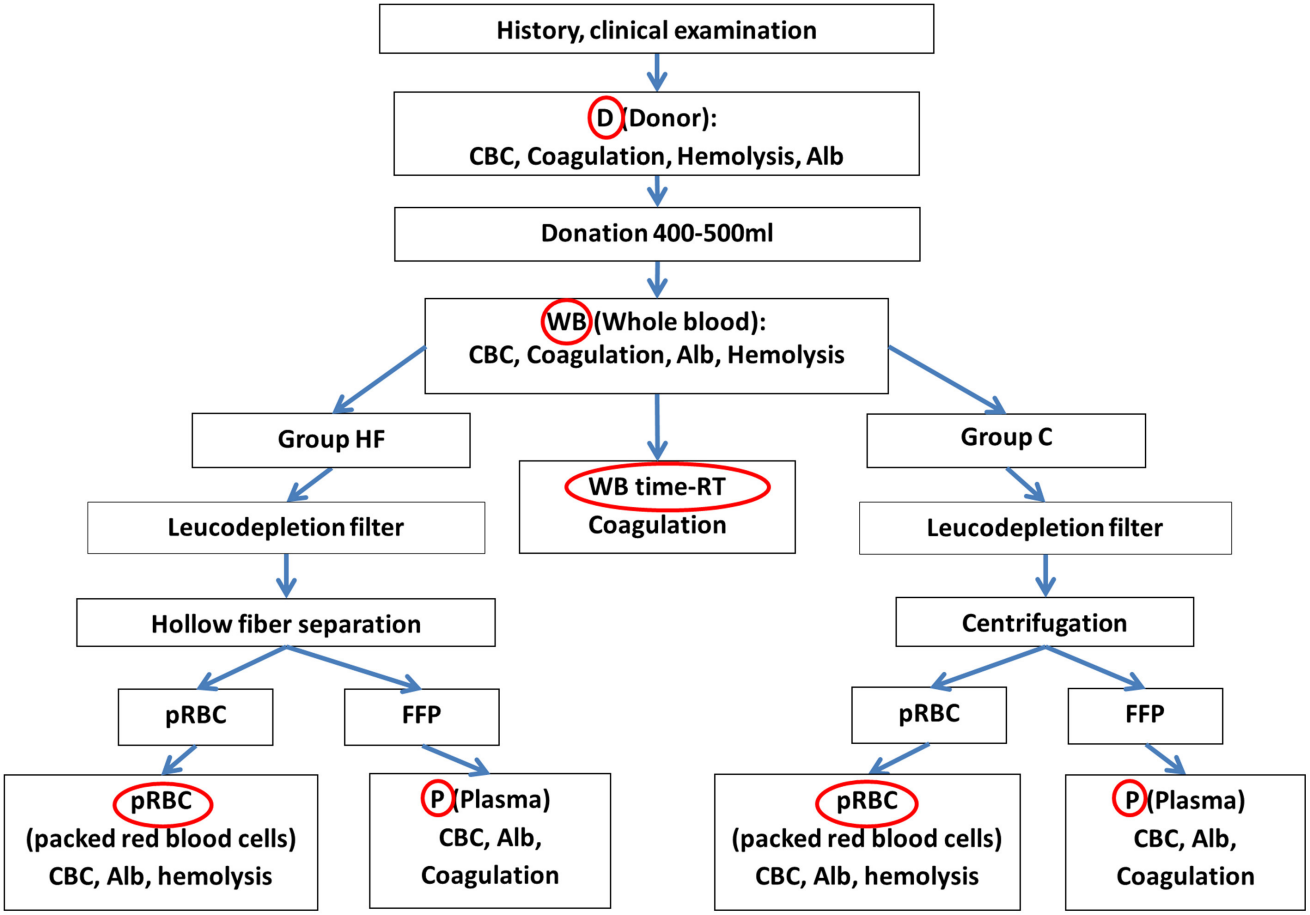
Flow chart representing all steps of the separation process and the origin of sample (red circles) as well as the evaluated parameters. Samples were taken from the donor (D), the whole blood bag (WB) prior to the separation process as well as the packed red blood cell bag (pRBC) and the plasma bag (P). The sole impact of time at room temperature (time-RT) during the separation process was evaluated with a subset of specimens from the WB which was centrifuged and frozen at the end of the procedure (WB time-RT). Alb, albumin; CBC, complete blood cell count including the hematocrit value, red blood cell count, hemoglobin concentration, platelet count, white blood cell count; Coagulation, coagulation parameters including coagulation times PT, aPTT, fibrinogen concentration, activity of antithrombin and factors VIII, V, and von Willebrand factor. FFP, fresh frozen plasma; hemolysis, parameters reflecting hemolysis including concentrations of phosphate, lactate dehydrogenase activity.

The exact procedure of sampling is shown in Figure 3. As seen in the figure and described previously, samples from the donor (D) were obtained for routine hematological and clinical chemical analysis. The results of the CBC, parameters reflecting hemolysis, the albumin concentration as well as the coagulation analysis served for comparison with the results obtained later from the respective blood bags.

All samples taken from the blood bags later on were transferred to 1.3 ml plain tubes. After blood donation, 15 ml of whole blood was aseptically drawn out of the collection bag (WB) and transferred into 10 aliquots for performance of the same analyses as in the blood donor. Six of Ten aliquots (named WB, Figure 3) were directly used for the same analyses as the samples directly taken from the donor (D) to assess the sole impact of anticoagulant in the WB bag. The remainder four of these 10 specimens (named WB time-RT) were left at room temperature during the separation process and served to assess the sole impact of time and room temperature (time-RT) during the process of blood separation on coagulation parameters. Directly at the end of the separation process, plasma was harvested, and kept frozen at −80°C until coagulation analysis.

After the separation process, 8 ml plasma were aseptically drawn from all (HF & C) plasma bags (P), as well as 5 ml erythrocyte concentrate from the respective pRBCs (compare Figure 3). Quality of the plasma was evaluated by assessing the CBC, the albumin concentration and coagulation parameters. Quality of the erythrocyte concentrate was assessed with a CBC, the albumin concentration and parameter reflecting hemolysis.

Hematological analysis was performed immediately after sampling. The remainder specimens were centrifuged as described before and frozen at −80°C until analysis.

After collecting samples, all bags were sealed. The pRBCs were kept refrigerated at 4°C and the plasma bags frozen at −30°C, respectively.

### Laboratory Examination

Hematological examination was performed within 4 h after sampling with a commercial automatic laser-based veterinary hematology analyzer^12^. CBC included hematocrit value (Hct), hemoglobin (hgb), red blood cell (RBC) count, white blood cell (WBC), and platelet count (Plt). Moreover, erythrocyte indices were assessed including the mean corpuscular volume (MCV), the cell hemoglobin concentration mean (CHCM) consistent with the mean hemoglobin concentration measured in every erythrocyte, and the mean corpuscular hemoglobin concentration (MCHC), i.e., the mean hemoglobin concentration calculated by the analyzer from the erythrocyte count and the total hemoglobin concentration.

To quantify the degree of hemolysis due to separation process, hemolysis in percent was calculated by us as reported previously using the Hct value, the total hemoglobin concentration (hgb_total_) and the free hemoglobin concentration (hgb_free_) in the blood plasma (25):

$$\text{Hemolysis }(\text{in}\%)\text{ = }(\text{1}-\text{Hct})\times ({\text{hgb}}_{\text{free}}{\text{/hgb}}_{\text{total}})\times \text{100}.$$

Hgb_total_ was measured with the hematology analyzer^12^. Hgb_free_ was calculated by subtracting the cellular hemoglobin concentration, i.e., the hemoglobin within the erythrocytes (hgb_cell_) that is not affected by hemolysis from hgb_total_. Hgb_cell_ was calculated by reversing the formula used to calculate the MCHC for the CHCM (hgb_cell_ = CHCM × RBC × MCV/1,000) (26). If hgb_total_ was lower than calculated hgb_cell_ due to the imprecision of the methods and multiple calculation steps, a slightly negative result was obtained for hgb_free_ that was censored as “zero.”

Biochemical analysis included measurement of the albumin concentration and of parameters indicating potential hemolysis, i.e., concentrations Phos and lactate dehydrogenase (LDH) activity. LDH and albumin were assessed with a commercial clinical chemistry benchtop analyzer^13^.

Coagulation analysis was performed as batch measurement within 4 months after sampling. Samples were allowed to thaw in a 37°C waterbath^14^ and were centrifuged again for 10 min at 800 G.

Coagulation analysis included measurement of prothrombin time (PT), activated partial thromboplastin time (aPTT), fibrinogen concentration, antithrombin (AT), and factor VIII activity (FVIII) measured with a commercial viscoelastic-based mechanical detection system^15^ as described previously (27).

Reference intervals have been established and published previously for these methods (27) and were used for comparison with the obtained results. As for measurement of FVIII activity, FV activity was assessed using the automated coagulation analyzer ^16^ and the canine pool plasma prepared for the previous study (27). A commercial human assay consisting of a FV-deficient plasma substrate was used, whereby the default setting for dilution of the sample was 1:20 and a modified PT was measured. Results were compared with a calibration curve prepared by serial stepwise dilution of canine pool plasma in the dilution steps 1:40: 1:60; 1:80, and 1:100, respectively.

Von Willebrand factor (vWF) activity was measured at an external laboratory^16^ using immunoturbidometry with a commercial analyzer^17^. Samples were shipped frozen to the laboratory.

### Quality Criteria

Blood products were reviewed afterwards if they fulfilled (1) german national guidelines for the use of blood products in the veterinary field (17) and (2) the European human guidelines (16).

For FFP, the German veterinary guideline for blood products (17) state that at least 50% of the initial FVIII activity should remain after freezing and thawing in the FFP. Leukoreduction is not explicitly requested in the guidelines but there should be <1 × 10^9^ WBC/l, <30 × 10^9^/l thrombocytes, and <3 × 10^9^/l erythrocytes remaining in the plasma bag (compare **Table 5**). In contrast, the human guidelines in Europe request a WBC of <0.1 × 10^6^ / Unit for leukodepleted FFP (16) (**Table 5**). There should be not less than an average of 70 IU FVIII per 100 mL (after freezing and thawing), RBCs < 6.0 × 10^9^/L and Plt < 50 × 10^9^/L per unit.

Based on the German veterinary guideline for blood products (17), a Hct value of 0.5 – 0.75 l/l, a hgb concentration of 17 g/dl, and <0.8% of hemolysis at the end of storage should be achieved for the pRBC (compare **Table 5**). The human European guideline requests a Hct of 0.5–0.7 l/l and a minimum of 40 g hgb/unit and <1 × 10^6^ WBC / unit in leukodepleted pRBC as well as sterility and absence of discolorisation (compare **Table 5**).

### Statistical Analysis

For all statistical analyses, a commercially available software^18^ was used.

To verify the assumption of normality, the Shapiro-Wilk test was used. Depending on the results, either unpaired *t*-test or Mann-Whitney U test was applied to compare differences between the blood products (pRBC and FFP) depending on the method of separation (HF vs. C). The impact of group (HF vs. C) and step of preparing the blood product (origin of sample) was assessed with a two-way repeated measures analysis of variance (ANOVA), which was followed by two post-tests, whereby a correction for multiple comparisons was performed automatically by the software. The Sidak's multiple comparison test was done to evaluate the group effect for each obtained type of sample and blood product (the within column-effect of the statistical table). A possible effect of the procedure of blood separation within each group was investigated with a Tukey's multiple comparison test (the within-row effect of the statistical table).

To evaluate differences in the proportions of blood products fulfilling quality criteria in both groups, a Fisher's exact test was used.

*P*-values were corrected according to the Bonferroni-Holm method to reduce the probability of a type 1 error (28). Corrections were made seperately for each type of statistical question listed in Tables 1–**6** as well as for each origin of sample (D, WB, P, pRBC given in Tables 2, 3) and guideline, respectively (given in **Tables 5**, **6**). For the two-way ANOVA test, *p*-values obtained for the factors “origin of sample,” “separation method,” and “interaction” between both factors, were corrected separately with the Bonferroni-Holm method.

**Table 1:** Comparison of clinical data, donated blood volume, and separation time for group HF and C.

**Parameter (unit)**	**HF**	**C**	**p-value**
Age (years)	4	5	0.63
	[2–6]	[1–6]	
Body weight (kg)	37.5	35.7	0.95
	[20.1–56.6]	[25–67]	
Donated volume (ml)	442	431	0.099
	[406–496]	[379–493]	
Separation time (min)	57	67	0.19
	[41–180]	[50–135]	

*Data are presented as median with range (in brackets). C, blood products obtained with the centrifuge; HF, blood products obtained with the hollow-fiber system. P < 0.05 are considered significant*.

**Table 2:** Gender and castration/neuter status of the donors in both groups: hollow-fiber system (HF) or centrifuge (C).

**Gender**	**HF**	**C**
Female spayed	1/16	3/15
Female	3/16	2/15
Male	8/16	4/15
Male neutered	4/16	6/15

**Table 3:** Hematological variables, albumin concentration, and von Willebrand Factor activity in samples obtained during different steps of the separation process performed with the centrifuge (C) or the hollow-fiber system (HF).

**Parameter (unit)**	**D**			**WB**			***P***			**pRBC**			**Reference interval**
	**HF**	**C**	***p*-value**	**HF**	**C**	***p*-value**	**HF**	**C**	***p-*value**	**HF**	**C**	***p*-value**	
Hct l/l	0.48	0.5	0.39	0.41	0.42	0.79	0	0	>0.99	0.57	0.62	0.0776	0.39–0.56
	[0.41–0.55]	[0.41–0.57]		[0.36–0.47]	[0.34–0.54]		[0]	[0]		[0.53–0.69]	[0.55–0.67]		
Hgb mmol/l	10.1	10.4	0.60	8.5	8.6	0.62	0	0	>0.99	11.6	12.7	0.0791	8.1–12.2
	[8.4–11.2]	[8.3–12.1]		[7.2–9.3]	[6.8–10.8]		[0–0.1]	[0–0.5]		[10.5–14.5]	[11.2–13.6]		
RBC 10^12^/l	6.99	7.16	0.70	5.97	6.07	0.95	0	0	>0.99	8.045	8.93	0.1308	5.64–8.3
	[6.19–7.78]	[5.74–8.23]		[5.17–6.57]	[4.78–7.48]		[0–0.01]	[0–0.02]		[7.32–9.95]	[7.56–9.59]		
MCV fl	69.1	69.6	>0.99	70.2	70.6	>0.99	66.9	67.1	0.064	71.2	71.4	>0.99	62.61–73.50
	[66–72.3]	[65.5–75.9]		[66.7–74.3]	[66.5–77.2]		[0–88.3]	[46.8–83.3]		[68.3–75.9]	[67.9–77.9]		
MCHC mmol/l	21.1	21.22	>0.99	20.44	20.65	>0.99	0	0	0.13	20.56 (33.12)	20.49 (33.01)	>0.99	20.82–23.53
	[20.37–22.02]	[20.45–21.85]		[19.59–21.35]	[19.98–26.71]		[0–78.67]	[0]		[19.75–21.76]	[19.91–21.35]		
CHCM mmol/l	21.02	20.94	>0.99	20.43	20.58	>0.99	20.42	20.85	>0.99	19.94	20.03	>0.99	20.82–23.53
	[20.58–21.49]	[19.98–21.49]		[20.04–21.12]	[19.74–21.04]		[0–24.95]	[0–22.46]		[19.5–20.59]	[19.4–20.59]		
Plt 10^9^/l	246.5	207	>0.99	209.5	171	>0.99	2	3	>0.99	3	4	0.98	150–500
	[126–329]	[156–511]		[49–285]	[49–391]		[0–8]	[0–18]		[0–11]	[2–64]		
WBC 10^9^/l	8.69	8.2	0.77	6.86	6.67	0.97	0.01	0.01	>0.99	0.01	0.05	>0.99	5–48–13.74
	[5.03–12.48]	[5.02–15.58]		[4.22–9.69]	[3.99–14]		[0–0.19]	[0–0.65]		[0–0.01]	[0.01–0.65]		
Alb g/l	30.1	30.2	0.84	24	24.9	0.62	**21.65**	**23.5**	**0.0162**	**6.9**	**4.5**	**0.0018**	29.6–37.01
	[27.3–33.9]	[28–34.1]		[22–29.3]	[21.7–30.7]		[19–25.1]	[21.5–27.6]		[4.1–11.6]	[3.4–8.7]		
vWF %	136	111	0.5363	123.5	75	0.1208	101	69	0.9681	109	63	0.1903	50–180
	[55–212]	[43–186]		[51–189]	[9–140]		[37–177]	[34–214]		[54–188]	[11–217]		

*Sample were obtained directly from the donor (D), the whole blood bag (WB)—and after finishing blood separation—from the plasma bag (P) and the packed red blood cell bag containing erythrocyte concentrate (pRBC). Results are given as median and range (in squared brackets) in SI. C, blood products obtained with the centrifuge; HF, blood products obtained with the hollow-fiber system; D, blood samples from blood donor prior donation; WB, blood samples from Whole blood bags after donation; P, plasma samples from plasma bag after separation process; pRBC, blood samples obtained from packed red blood cell bag after separation; Hct, hematocrit value; Hgb, hemoglobin concentration; RBC, Red Blood Cell Count; MCV, mean corpuscular volume; MCHC, Mean Corpuscular Hemoglobin Concentration; CHCM, Cell Hemoglobin Concentration Mean; Plt, platelet count; WBC, white blood cell count; Alb, albumin concentration; vWF, von Willebrand factor activity. P < 0.05 were considered significant and printed bold*.

For all statistical analyses, level of significance was set at *p* < 0.05.

## Results

During the sampling period, 32 non-Greyhound dogs were presented for blood donation. One of 32 dogs assigned to the HF group had to be excluded due to severely lipemic plasma (triglyceride 4.4 mmol/l), which markedly impaired separation process. Failure of separation was characterized by unequal distribution of weight between plasma and erythrocytes (90 vs. 314 g) and remaining 98 g within the filter.

Thus, a total of 31 dogs were enrolled in the study.

Overall, 15/31 whole blood bags were processed with centrifugation protocol, whereas 16/31 bags were processed with the gravity-driven HF system.

### Donations

All donations went uneventfully and were finished within a median 6 min (range 5–10 min, Table 1).

As shown in Table 1, age, body weight, and the donated volume did not differ significantly between the two groups. The sex, castration, and neuter status of the donor dogs are shown in Table 2.

### Separation Process

The use of the novel gravity-driven HF system was easy, less (working) time consuming than the centrifuge as no preparation of the centrifugation cup, no taring of the centrifugation cups, no unloading and mechanical expression is necessary. It was well-appreciated by technicians and veterinarians. Familiarity was reached after a few separation procedures. There was no difference in separation time between the groups, but the time required for two of 31 separation processes using the HF exceeded the time frame of 90 min recommended by the manufacturer for a separation process (13, 30). Both bags contained a relatively high volume of 496 and 483 ml, respectively, which was at the upper range of the overall donated volume (Table 1).

### Quality of the FFP

**Figure 4:**
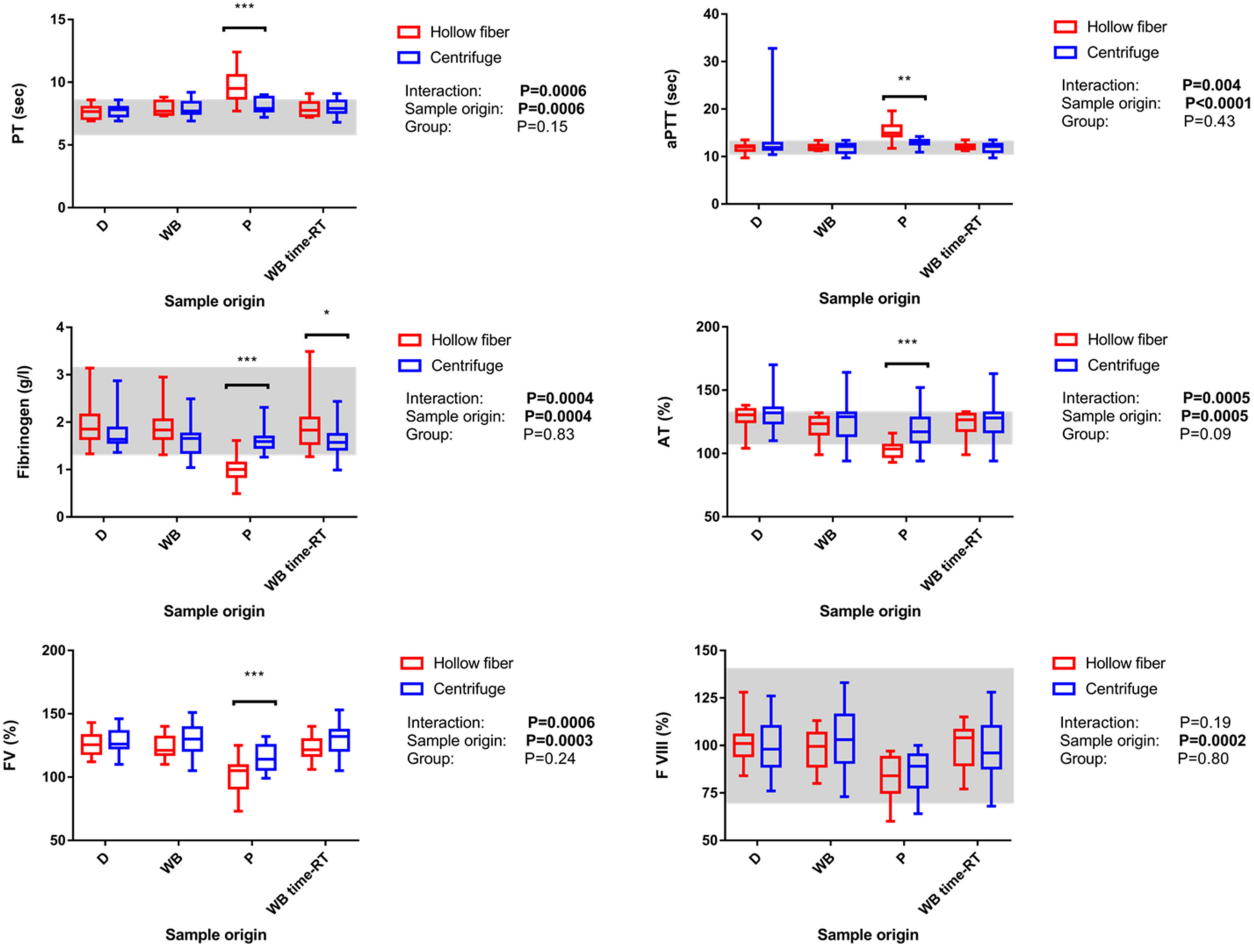
Impact of origin of sample reflecting the different steps of the blood separation process and the system used for blood separation (i.e., centrifuge or the hollow-fiber system) on coagulation parameters as well as interaction between the factors “origin of sample” and “system” Data is shown as box-and-whisker diagrams. The central line represents the median, the box the 25 and 75% percentile, and the whiskers are consistent with the range. The reference interval is indicated as gray area. Significant pairwise comparisons were marked with ^*^-^***^; whereby ^*^P < 0.05; ^**^P < 0.01, and ^***^P < 0.001, respectively. D, blood samples from blood donor prior donation; WB, blood samples from Whole blood bags after donation; P, plasma samples from plasma bag after separation process; WB time-RT, sample taken from the whole blood bag prior to the blood separation process to assess the sole impact of time at room temperature; PT, prothrombin time, aPTT, activated partial thromboplastin time; AT, antithrombin activity; FV, factor V activity; FVIII, factor VIII activity.

After the separation process, PT and aPTT assessed in the plasma bag (P) were significantly higher in the HF group compared to C, while fibrinogen concentration as well as FV and AT activity were significantly lower (Figure 4). After separation, the vWF activity was not significantly different between the two groups (Table 3). Comparing WB time-RT and P, there was no impact of time and therefore sample aging during the separation process on the results.

Following the separation process, fibrinogen concentration assessed in P prepared with the HF system decreased in all but 1/16 HF plasma units below the lower limit of the reference interval. Only 12/16 HF plasma bags contained 50% or more of initial fibrinogen concentration after freezing and thawing (Figure 4).

After leukodepletion and separation, a significant decrease of FVIII activity could be observed in both groups, whereas FVIII remained high in WB time-RT in both groups, i.e. there was no impact of sample aging on the results (Figure 4). In both groups, FVIII remained above 50% of initial activity in all samples (**Table 5**).

In HF, the separation process resulted in a significantly higher decline of albumin concentration compared to C (Table 3).

### Quality of the Erythrocyte Concentrate (pRBC)

The hematocrit value was not—significantly higher in pRBC in group C compared to the HF group. Similar results could be obtained for the hgb concentration (Table 3). In D or WB samples, no significant difference between group HF and C for the Hct, hgb concentration or RBCs was observed.

Erythrocyte indices (MCV, MCHC, and CHCM) in pRBC did not differ between the two study groups (Table 3).

As expected, leukodepletion process resulted in a significant decrease in Plt and WBC counts from baseline (WB) cell counts (Table 3).

There was no significant difference between the two groups at baseline (D and WB) or after the separation process (in P and pRBC) in Plt or WBC count.

Moreover, separation method did not have an impact on Phos concentration, LDH activity as well as calculated %hemolysis in the samples obtained from the D, WB, and pRBC (Table 4).

**Table 4:** Impact of blood separation process on median and range of parameters reflecting hemolysis in samples obtained from the donor (D), the whole blood bag (WB), and the packed red blood cell bag (pRBC).

**Parameter (unit)**	**D**			**WB**			**pRBC**			**Reference interval**
	**HF**	**C**	***p*-value**	**HF**	**C**	***p*-value**	**HF**	**C**	***p*-value**	
Hemolysis (%)	0	0	N/A	0	0	N/A	0.29	0.47	N/A	<0.8^*^
	[0–1.7]	[0–1.6]		[0–0.2]	[0–3.418]		[0–1.8]	[0–1.9]		
Phos (mmol/l)	1.39	1.39	>0.99	3.71	3.56	0.81	0.95	0.76	0.34	0.79–2.1^#^
	[1.03–1.99]	[0.88–1.71]		[3.11–4.24]	[2.39–4.43]		[0.63–1.45]	[0.56–1.31]		
LDH (U/l)	25	24.5	>0.99	22.5	30	0.72	10	14	0.30	85.7–255^#^
	[10–69]	[2–64]		[1–60]	[1–160]		[1–35]	[1–261]		

*Phos, phosphate concentration and LDH, Laktatedehydrogenase activity. P < 0.05 considered significant; % hemolysis calculated as follows: [(1-hematocrit value) × 100 × free hemoglobin/total hemoglobin]*.

* *Bundesamt für Verbraucherschutz und Lebensmittelsicherheit (17)*.

# *Laboratory intern reference interval obtained in 56 dogs (27)*.

### Comparison With Guidelines Regulating Quality of Blood Products

As shown in Table 5, FVIII activity in the FFP remained high enough to fulfill national veterinary guidelines, but RBC counts were exceeding the allowed cut-off values in more than 40% of plasma products obtained with both separation methods. The maximally allowed WBC counts of <1 × 10^9^/l requested by the German veterinary guideline for blood products could be fulfilled in all plasma bags. On the other hand, the WBC count of <0.1 × 10^9^/l requested by human European guidelines could not be achieved in some FFP in both groups (16).

**Table 5:** Impact of the method of the separation process on quality of the obtained fresh frozen plasma (FFP) as reflected by FVIII activity and the remainder number of WBC, RBC, and Plts.

	**FFP**			German Veterinary Guideline FFP^*^	European human Guideline FFP^**^	Fulfilling German Veterinary criteria^*^			Fulfilling European human criteria^**^		
	**HF**	**C**	***p*-value**			**HF**	**C**	***p*-value**	**HF**	**C**	***p*-value**
Remaining activity of FVIII in % compared to WB	87	86	0.97	>50%	>70 U/100 ml^#^	16/16 (100%)	15/15 100%	>0.99	N/A	N/A	N/A
	[72.28–96]	[65.31–112.3]									
RBC 10^9^/l	0	0	>0.99	<3 × 10^9^/l	<6 × 10^9^/l	9/16 (56.3%)	9/15 (60%)	0.67	9/16 (56.3%)	9/15 (60%)	0.67
	[0–10]	[0–20]									
Plt 10^9^/l	2	3	0.98	<30 × 10^9^/l	<50 × 10^9^/l	16/16 (100%)	15/15 (100%)	1.0	16/16 (100%)	15/15 (100%)	>0.99
	[0–8]	[0–18]									
WBC 10^9^/l	0.01	0.05	0.99	<1 × 10^9^/l	<0.1 × 10^9^/l	16/16 (100%)	15/15 (100%)	1.0	13/16 (81%)	14/15 (93%)	0.057
	[0–0.01]	[0.01–0.65]									

*Reported are median results are reported and the number of plasma bags fulfilling human and veterinary quality requirements. Hct, hematocrit value; Hgb, hemoglobin concentration; N/A, not applicable; Plt, platelets; WBC, white blood cell count; p < 0.05 were considered statistically significant and depicted in bold letters*.

* *Bundesamt für Verbraucherschutz und Lebensmittelsicherheit (17)*.

** *European Directorate for the Quality of Medicines & HealthCare (16)*.

# *The activity of 4.9 units of human FVIII is consistent with 1 mg (29); 1 unit/kg induces an 2% increase in FVIII activity in people*.

Regarding quality of the pRBC (Table 6), both separation methods resulted in adequate quality for obtained concentrations of RBCs, hgb, and Htc value. However, irrespective of the separation method, approximately one third of pRBC bags of both groups exceeded the requested cut-off value of 0.8% for hemolysis. Moreover, the degree of achieved leukodepletion was not sufficient for any pRBC prepared here when compared with human European guidelines.

**Table 6:** Impact of the method of the separation process on quality of the packed red blood cell bag (pRBCs) as reflected by erythrocyte parameters, remainder WBC, and %hemolysis.

**Parameter (unit)**	**pRBC**			German Veterinary Guideline pRBC^*^	European human Guideline pRBC^**^	Proportion fulfilling German Veterinary criteria^*^			Proportion fulfilling European human criteria^**^		
	**HF**	**C**	***p*-value**			**HF**	**C**	***p*-value**	**HF**	**C**	***p*-value**
Hct l/l	0.57	0.62	0.0776	0.5–0.75	0.50–0.70	16/16 (100%)	15/15 (100%)	>0.99	16/16 (100%)	15/15 100%	>0.99
	[0.53–0.62]	[0.55–0.67]									
Hgb mmol/l	11.6	12.7	0.0791	>10.5 (>17)	N/A	16/16 (100%)	15/15 (100%)	>0.99	N/A	N/A	N/A
	[10.5–14.5]	[11.2–13.6]									
Hemoglobin g/unit	55.27	59.54	0.72	N/A	>40 g/Unit	N/A	N/A	N/A	16/16 (100%)	15/15 (100%)	>0.99
	[43.3–71.01]	[45.04–72.3]									
WBC × 10^6^/Unit	19.94	20.03	>0.99	N/A	<1 × 10^6^/Unit	N/A	N/A	N/A	0/16 (0%)	0/15 (0%)	>0.99
	[19.5–20.59]	[19.4–20.59]									
Hemolysis %	0.29	0.47	N/A	<0.8%	<0.8%	11/16 (68%)	10/15 (66%)	0.88	11/16 (69%)	10/15 (66%)	0.88
	[0–1.8]	[0–1.9]									

*Reported are median results and the number of pRBCs fulfilling human and veterinary quality requirements. C, blood products obtained with the centrifuge; HF, blood products obtained with the hollow-fiber system; pRBC, blood samples from packed red blood cell bag after separation; Hct, hematocrit; Hgb, hemoglobin concentration; WBC, White blood cell count, % hemolysis calculated as follows: [(1-hematocrit value) × 100 × free hemoglobin/total hemoglobin], N/A, not applicable. P < 0.05 are considered significant and depicted in bold letters*.

* *Bundesamt für Verbraucherschutz und Lebensmittelsicherheit (17)*.

** *European Directorate for the Quality of Medicines & HealthCare (16)*.

## Discussion

To the authors' knowledge, this is the first study in veterinary medicine evaluating quality of blood product after the separation process using a HF system.

Due to its ease of use, HF separation would be a fast and feasible tool for veterinary clinics and practices with limited space and resources to use a centrifuge. Theoretically, smaller clinics and veterinary practices might consider using whole blood instead of prepared blood products, however, storage of whole blood at 4°C results in significantly higher decrease of coagulation factor activity than in frozen plasma (31). Moreover, plasma rather than whole blood transfusion is recommended in non-anemic patients only requiring coagulation factors or protein to avoid RBC, volume or iron overload and vice versa dogs with e.g. immune mediated hemolytic anemia with normo- or hypervolemia are at risk to experience Transfusion associated circulatory overload (TACO) (32, 33).

Separation time was similar for both methods evaluated here. However, in accordance with previous investigationevaluating the preparation of human blood products, labor intensity was markedly lowerof the separation process compared to centrifugation (12), as preparation and taring of the centrifugation cup as well as unloading and mechanical expression of the plasma bag is not necessary when using the HF.

However, quality of blood products was impaired when using HF separation process as reflected by a significant decrease in coagulation factor activity and fibrinogen concentration finally leading to increased coagulation times. Fibrinogen concentration was markedly decreased compared to the traditional centrifugation method. In accordance with our results, previous studies in people evaluating HF systems of the same composition, reported lower FVIII activity and fibrinogen concentration (14). On the other hand, acceptable FVIII (69–79% of initial activity present after 1 year of storage) activity has been demonstrated for human FFP as required by the Council of Europe (16) despite a decline in plasma protein content and coagulation factor activity due to postulated absorption by the HF system (12). The utilization of polyethylene instead of polyethersulfone fibers in the filter system showed no obvious impairment of coagulation factor activity in a previous study (34). However, none of the previous studies performed a direct comparison between the two separation methods as we did. A recent study separating human whole blood with a similar device as we did already recognized an increase in PT, aPTT and decrease in fibrinogen concentration, FV, FVII and FVIII activity which is in accordance with our results (8).

In our study, FVIII activity was above 50% of initial activity in both groups. As in people, no further assessment of coagulation factor activity or concentration is demanded in the German veterinary guideline for blood products (17). Nevertheless, we measured activities of FV and vWF as well as the fibrinogen concentration and the coagulation times PT and aPTT to enable a comparison with previous veterinary studies (35, 36). The requirements of European human guidelines could only be partially fulfilled for plasma quality as RBC counts were too high in both groups. Therefore, in regard of fulfilling quality requirements, plasma obtained with the HF group is of lower quality than plasma generated by centrifugation. However, further studies evaluating the clinical utility of plasma products obtained with HF would be needed to demonstrate the clinical significance, i.e., the impact of lower coagulation factor activity or fibrinogen concentrations on patient outcome. Human studies suggest that the degree of fibrinogen substitution might be clinically relevant (37) in the way that that the transfusion of plasma containing high concentrations of fibrinogen is superior to lower concentration products in surgical and massive trauma patients.

The exceedance of separation time of 90 min in two donors can be considered as separation failure as done in a previous study (13). However, the rationale behind such limited time frame given for separation procedure remains unclear as sample aging due to the separation did not affect coagulation results as demonstrated here. The reason for observing prolonged separation time in the 2 blood bags seen here has not been clarified. Theoretically, leukocyte count and donated blood volume causing exhaustion of the HF might affect separation time. Leukocyte counts, however, were not increased in these two P and pRBC bags, so that failure of leukodepletion can be excluded as potentially explanation. A volume of the blood bag not exceeding 450 ± 50 ml has been recommended by the manufacturer (13, 30). It remains unclear if the leukodepletion filter itself, any clots within the leukodepletion filter or HF exhaustion caused the significant increase in separation time. In previous studies, separation times of 40 and 44 min were described using a not further specified Fresenius and a Pall leukodepletion filter (12, 14) similar to the filter used in this study. Studies not using any leukodepletion filter reported a separation time of 11 and 15 min although they used a HF system with similar fiber diameter (328 μm) as in our study but smaller pore size (0.2 μm) andlower wall thickness (30 μm) (11, 34).

Based on the European human guideline for quality of FFP and pRBC recommend a reduction to <1 × 10^6^ WBC per Unit (16), which could not be achieved here and in previous studies (38). Leukodepletion by buffy coat separation prior filtering through a leukodepletion filter might be more efficient, but is not possible in this system or study design (22, 39). It has been stated earlier that leukodepletion might be more efficient after storage for 2–4 h at 4°C (40).

A potential impact of implementing the leukocyte reduction filter on coagulation factor activity was taken into account while planning the study, as leukodepletion was done by the same filter for both groups prior separation process. Decrease in FVIII in both groups can be atrributed to leukodepletion in both groups, as in WB time-RT FVIII activity remained high which excludes time as effector for the decline. It has been shown previously that the use of the same filter as applied in our study (WBF3 Leukodepletion filter)^5^ causes a significant decline in FVIII activity and fibrinogen concentration (41). Performing the step of leukodepletion after HF separation only for the pRBC bag might positively influence plasma product quality, at least regarding FVIII and fibrinogen.

The crystalloid infusion during the donation process might have some hemodilutional effect on the donated blood and thus have a potential impact on the laboratory parameters assessed here. However, as both groups received the same infusion rate per kg body weight, they experienced the same degree of hemodilution, so that the effect of the method of blood separation, i.e., the group effect can be still compared. The same applies for the dilutional effect ofthe anticoagulant CPD that has a potential impact on the assessed parameters but does not affect the group effect as it is similar in both HF and C.

We appreciate some limitations of our study.

First, we did not use a special technique of randomization so that a selection bias cannot be entirely ruled out. However, the groups did not differ significantly prior to the separation process, a major selection bias appears to be unlikely.

Moreover, our study was focussing on the short-term effect of the method of separation rather than its potential impact on quality of blood products after storage, i.e. the long term effect. Thus, quality of erythrocytes at the end of storage as reflected by the degree of hemolysis in pRBC at the end has not been evaluated as required in the German Veterinary guidelines (17). A previous human study (13) showed, that hemolysis starts to increase after day 22 of storage reaching >0.8% hemolysis on day 29 demanding further research for canine pRBC. Hemolysis has been evaluated for leukodepleted and non-leukodepleted pRBC in veterinary and human literature showing some evidence of increased hemolysis depending on filter type and material (38, 40, 42–44) emphasizing the need for further studies.

We also did not evaluate pRBC quality over time which would have included assessment of 2.3-diphosphoglyceric acid, LDH, K (in humans) and free hemoglobin concentrations as well as bacterial cultures after expiring of the pRBC bags. In people, erythrocytes are of high potassium content and potassium concentration in blood products is therefore a suitable marker of hemolysis and thus the quality of blood products. In contrast, canine erythrocytes are of low potassium content except for some Japanese dog breeds and are therefore not reliably reflecting the degree of hemolysis. Thus, potassium concentration has not been used here to assess the quality of the blood products.

As done previously, plasma quality could be further assessed with thromboelastography (36), to improve predictability of coagulation factor activity *in vitro* and to estimate patients response to plasma therapy.

While assessment of quality was based on *in vitro* analyses, its clinical utility, i.e., improvement of coagulation reaction in the patient has not been evaluated.

## Conclusion

Using the HF blood separation system provides blood products with impaired quality especially regarding coagulation parameters when compared to the centrifuge, however, the clinical significance of the finding still has to be elucidated.

Nevertheless, the HF is an easy to use alternative, which is interesting for veterinary clinics or practices with limited space and resources to buy a centrifuge.

## Ethics Statement

This study was carried out in accordance with the recommendations of Bundesamt für Verbraucherschutz und Lebensmittelsicherheit, 2011. Leitlinien zur Gewinnung, Lagerung, Transport und Verabreichung von Blut und Blutprodukten im Veterinärbereich, Germany. (Federal Office for Consumer Protection and Food Safety, 2011. Guidelines for the collection, storage, transport and administration of blood and blood products in the veterinary field, Germany).

The blood donation program was ethically approved by the regional council (No. V 54 - 19 c 20 15 h 02 GI 18/7 No. A 24/2017) and written owner's consent was given. The blood products were implemented in patient care.

## Author Contributions

EMH, NB, AM, EH, and HL designed the study. HL, NB, EMH, and EH drafted the manuscript. HL and EMH collected data and all authors contributed to its review.

### Conflict of Interest Statement

The authors declare that the research was conducted in the absence of any commercial or financial relationships that could be construed as a potential conflict of interest.

## Footnotes

^1^KLINIJECT® venous catheter, KLINIKA Medical GmbH, Usingen, Germany.

^2^Quick Test DEA 1, Alvedia, Limonest, France.

^3^Mikro 120, Andreas Hettich GmbH & Co.KG, Tuttlingen, Germany.

^4^3M^TM^ Blood Separation System – Blood-bag system, 3M Deutschland GmbH, Neuss, Germany (former name PlasmaXpress Membrana, Germany).

^5^Leukotrap WBF3 (Leukodepletion filter), Haemonetics Corperation, Baintree, MA, USA (former Pall, Pall Biomedical, Portsmouth, UK).

^6^Blood mix scale BioTrans MW-5001, Möller Feinmechanik GmbH & Co, Fulda, Germany.

^7^Sterofundin® ISO 1/1 E ISO, B. Braun Melsungen AG, Melsungen, Germany.

^8^Volumetric infusion pump, Caesarea Industrial Park, Caesarea, Israel.

^9^3M^TM^ Blood Separation System – Hollow Fiber MicroPES, 3M Deutschland GmbH, Neuss, Germany (former name PlasmaXpress Membrana, Germany).

^10^ROTANTA 460 R, Andreas Hettich GmbH & Co.KG, Tuttlingen, Germany.

^11^Manual Plasma Extractor, unknown, unknown.

^12^ADVIA2120™, Siemens Healthcare GmbH, Erlangen, Germany.

^13^ABX Pentra 400, HORIBA ABX SAS, Montpellier, France.

^14^Wasserbad, GFL Gesellschaft für Labortechnik mbH, Burgwedel, Germany.

^15^STA Compact®, Diagnostica Stago S.A.S., Asnières-sur-Seine, France.

^16^LABOKLIN GmbH & Co. KG, Labor für klinische Diagnostik, Bad Kissingen, Deutschland.

^17^Immunoturbidometry, Cobas 8000 c701, Roche Germany Holding GmbH, Germany.

^18^GraphPad Prism Version 7.0, Graph Pad Software Inc., San Diego, USA.

## Abbreviations

Alb: albumin
aPTT: activated partial thromboplastin time
AT: antithrombin (formerly antithrombin III)
C: centrifugation
CBC: Complete Blood Count
CHCM: Cell Hemoglobin Concentration Mean
CPD: Citrat-Phosphate-Dextrose
D: samples prior donation
FFP: fresh frozen plasma
FV: factor V activity
FVIII: factor VIII activity
Hct: hematocrit value
hgb: hemoglobin concentration
HF: hollow-fiber system
K: potassium
LDH: lactate dehydrogenase
MCHC: Mean Corpuscular Hemoglobin Concentration
MCV: mean corpuscular Volume
P: plasma bag
Phos: phosphate
Plt: platelets
pRBC: packed red blood cell bag
PT: prothrombin time
RBC: red blood cells
SAG-M: Saline Adenine Glucose-Mannitol-Solution
vWF: von Willebrand Factor activity
WB: whole blood bag
WBC: white blood cells
WB time-RT: whole blood samples stored at room temperature for the time of the separation process.
